# Evaluation of Physical Activity Level and Related Factors in Pregnancy During the COVID-19 Period

**DOI:** 10.3389/ijph.2023.1605800

**Published:** 2023-05-05

**Authors:** Zeynep Meva Altaş, Nimet Emel Lüleci, Seyhan Hıdıroğlu

**Affiliations:** Department of Public Health, School of Medicine, Marmara University, Istanbul, Türkiye

**Keywords:** neighborhood, physical activity, pregnant women, pregnancy physical activity questionnaire, inactivity

## Abstract

**Objectives:** It was aimed to determine the level of physical activity and related factors in pregnant women.

**Methods:** The study is a mixed methods study. The participants are women applied to the pregnancy outpatient clinic of a hospital. The level of physical activity was assessed with the Pregnancy Physical Activity Questionnaire. Sociodemographic questions and seven questions of the International Physical Activity Environment Module were asked. Besides, in-depth interviews were conducted with 14 women.

**Results:** The study was conducted with 304 women. The median age was 29.0 (18.0–40.0) years. The mean total activity and sedentary activity scores were 195.8 ± 107.9 and 37.22 ± 31.08 MET-hours/week, respectively. Pregnant women were mostly involved in light-intensity and housework/caregiving activities. Most of the participants mentioned that they were less active than pre-pregnancy period. The most common reasons for being less active were weakness, fatigue, lack of time and complaints such as low back pain and nausea.

**Conclusion:** More than half of the pregnant women mentioned that they were less active during pregnancy. Thus, interventions should be planned to increase physical activity level of pregnant women.

## Introduction

Physical activity (PA) is one of the most important determinant of health in pregnancy. However; pregnant women tend to decrease physical activity levels with the effect of physiological and psychological changes [1]. The American College of Obstetricians and Gynecologysts ACOG states that women should acquire healthy habits before and during pregnancy [2].

There are many health benefits of PA. Women who are more active during pregnancy can adapt better to the changes related to pregnancy [3]. PA reduces risk of gestational diabetes, hypertension, low back pain and depression symptoms [3–6]. PA performed during pregnancy shortens the duration of the labor and reduces the stillbirth rate [7, 8].

Pregnant women should be encouraged and supported for regular physical activity. The ACOG recommends moderate-intensity physical activity for at least 150 min every week for uncomplicated pregnancies [2]. Unfortunately, less than 15% of women can perform the regular physical activity recommended for pregnant women [9]. Studies show that many women reduce their physical activity levels after conception compared to pre-pregnancy periods [10, 11]. This decrease is observed in the type, frequency and duration of PA [12]. In addition, studies reported that PA levels also changes during the pregnancy trimesters [13, 14].

The number of studies evaluating the effect of the measures taken during the pandemic on physical activity is low. In a qualitative study, pregnant women stated they were inactive during the pandemic period and gave up their daily routines such as walking [15]. Another study conducted in 2021; stated 56.3% of the women, 48.1% of the men were inactive [16]. Morover, there is an increase in conditions such as depression and anxiety in some women, and a decrease in physical activity as a result of isolation precautions during pregnancy or postpartum period [17, 18]. It can be said that pregnant women are negatively affected in terms of physical activity during the COVID-19 pandemic.

Since physical inactivity during pregnancy causes negative health outcomes, evaluation of the physical activity levels and the factors effecting physical activity levels in pregnant women are important. This study aims to determine the PA level of pregnant women who apply to the pregnancy outpatient clinic and examine the ractors that may effect the physical activity.

## Methods

### Study Design

The study is a mixed methods study with qualitative and quantitative parts. The population consists of women applied to the pregnancy follow up clinic of a hospital in Istanbul. Sample size was calculated as 294, as the rate of physical inactivity in pregnant women was 74.3% [19], the margin of error was 5%, and the confidence level was accepted as 95%. In the qualitative part of the study, a case study design was used. In-depth interviews were conducted with 14 pregnant women.

### Measures

Physical activity level of pregnant women was assessed with Pregnancy Physical Activity Scale (PPAQ) developed by Lisa Chasan-Taber et al. [20]. PPAQ’s Turkish validity and reliability was performed [21]. PPAQ determines the activity level of pregnant women based on the time of 32 activities (household, occupation, sports/exercise, transportation, inactivity). The amount of time is asked weekly or daily for each activity [21]. To calculate weekly energy expenditure; the duration of time spent in each activity is multiplied by specific intensities (MET values), and scores are expressed in MET-hours/week. One MET refers to the energy expenditure at rest [22]. The intensity of each activity is classified: Sedentary (≤1.5 MET), light (1.5–3.0 MET), moderate (3.0–6.0 MET), vigorous (>6.0 MET) [21]. Activity types are classified as: household/caregiving, occupational, sports/exercise activities. All scores of PPAQ (total score, activity intensity, activity type) are continous variables. There are no cut-off points. The higher the score obtained from the scale’s questions related to the activity type and activity intensity; the higher the time spent on that activity type or activity intensity.

The International Physical Activity Environment Module (IPAQ-E) is a tool for the assessment of environmental factors related to physcial activity [23, 24]. Seven questions from the IPAQ-E that are considered important for our study were asked in the questionnaire as independent variables in addition to sociodemographic questions (23 questions). IPAQ-E evaluates environmental features in the neighborhood, such as the suitability of the pavements for walking, the presence of free sports fields. Responders answer these questions as 3 different options: I agree, I do not know/I am not sure, I disagree. Dependent variables of the study were the total score and activity type and intensity scores of PPAQ scale, as mentioned above.

### Data Collection

A face-to-face questionnaire was applied to the pregnant women after their consent. Besides, consent was obtained from 14 women for in-depth interviews. Interviews were conducted via phone calls on an another day. Physical complaints in pregnancy and the effects of COVID-19, which we think may affect their physical activity were asked with semi-structured interview technique. The median duration of the interviews was 23.5 (20.0–34.0) minutes. Answers were audio recorded after the consent of women. These records were used for the study anonymously and never shared.

### Statistics

SPSS (Statistical Package for Social Sciences) for Windows 25.0 was used for statistical analysis. Median, minimum, maximum values, numbers (n), percentages (%) were used for descriptive data. In non-normally distrubuted data; Mann Whitney U and Kruskal Wallis tests were used. In normally distrubuted data, Student’s t-test and ANOVA were used. Spearman test was used for the comprasion of two numeric variables. Binary logistic regression test was applied for the multivariate analysis. Significance level was accepted as *p* < 0.05.

For the qualitative part; the audio records of the interviews were transcribed and transferred to digital media. Then content analysis was made. Codes and themes were determined. And direct quotations of women were included.

### Ethics

Ethics committee approval was received by the Clinical Research Ethics Committee of the relevant university (Date:02.04.2021, number: 09.2021.499).

## Results

### Descriptive Findings

Data of 304 pregnant women were evaluated. The median age was 29.0 (18.0–40.0) years. The percentages of women graduated from primary school, high school and university were 28.6%, 29.9%, 32.6%, respectively. Of the women 19.1% (*n* = 58) had chronic diseases. Median gestational week was 25.0 (5.0–36.0) weeks. Of the pregnant women, 20.4% (*n* = 62) had a miscarriage history and 18.1% (*n* = 37) had a high risk pregnancy before (Table 1).

**TABLE 1:** Sociodemographic characteristics and pregnancy-related conditions of the participants (Istanbul, Türkiye. 2023).

Sociodemographic characteristics	
Age, median (min-max)	29.0 (18–40)
Income (TL), median (min-max)	4,650.0 (2,800.0–40,000.0)
Education status, n (%)	
Illiterate	2 (0.7)
Literate	12 (3.9)
Primary education	87 (28.6)
High school	91 (29.9)
University	99 (32.6)
Master	13 (4.3)
Working status, n (%)	
Not working	215 (70.7)
Active working	64 (21.1)
On maternity leave	25 (8.2)
Presence of chronic disease, n (%)	
Yes	58 (19.1)
No	246 (80.9)
Pregnancy-related conditions	
Gestational week, median (min-max)	25.0 (5.0–36.0)
Number of pregnancies, median (min-max)	2.0 (1.0–7.0)
Number of children, median (min-max)	1.0 (0.0–6.0)
History of miscarriage/stillbirth, n (%)	
Miscarriage	62 (20.4)
Stillbirth	6 (2.0)
Both	4 (1.3)
No	232 (76.3)
Total	304 (100.0)
History of high-risk pregnancy, n (%)	
Yes	37 (18.1)
No	167 (81.9)
Total	204 (100.0)

The mean height, weight, BMI values were 162.04 ± 5.72 cm, 70.99 ± 13.33 kg, 27.04 ± 4.79 kg/m^2^, respectively. While the mean weight values before pregnancy were 64.53 ± 13.04 kg, the mean values for BMI before pregnancy were 24.59 ± 4.86 kg/m^2^.

### Evaluation of Physical Activity

The mean total activity score obtained from PPAQ was 195.8 ± 107.9 MET-hours/week. The sedentary activity median score was 27.5 (1.9–158.0) MET-hours/week. Median values of light, moderate, vigorous activity scores were 94.1 (0.0–282.5), 35.9 (0.0–327.6) and 0.0 (0.0–73.5) MET-hours/week, respectively. The median scores for household/caregiving, occupational, sports/exercise activities were 93.7 (0.0–470.8), 0.0 (0.0–245.0) and 6.6 (0.0–176.8) MET-hours/week, respectively (Table 2).

**TABLE 2:** Pregnancy Physical Activity scores (Istanbul, Türkiye. 2023).

PPAQ (MET-hours/week)	
Total activity, mean ± SD	195.84 ± 107.90
Activity intensity, median (min-max)	
Sedantary (<1.5 MET)	27.5 (1.9–158.0)
Lİght (1.5– < 3.0 MET)	94.1 (0–282.5)
Moderate (3.0–6.0 MET)	35.9 (0–327.6)
Vigorous (>6.0 MET)	0 (0–73.5)
Activity type, median (min-max)	
Household/Caregiving	93.7 (0–470.8)
Occupational	0 (0–245.0)
Sports/exercise	6.6 (0–176.8)

### Age, Income, BMI

A moderate positive correlation (r = 0.409, *p* < 0.001) was found between the occupational activities and income. Other correlations between age, income, BMI, and physical activity scores of pregnant women are shown in Table 3.

**TABLE 3:** Relationship of physical activity with age, income and Body Mass Index (Istanbul, Türkiye. 2023).

PPAQ (MET-hours/week)	Age	Income	Pre-pregnancy BMI
	Rho p	Rho p	Rho p
Total activity	0.014	0.118	−0.037
	0.807	0.039	0.523
Activity intensity			
Sedantary (<1.5 MET)	−0.148	0.331	−0.180
	0.010	<0.001	0.002
Light (1.5– < 3.0 MET)	0.077	−0.093	0.101
	0.179	0.105	0.080
Moderate (3.0–6.0 MET)	0.013	0.036	−0.043
	0.825	0.528	0.458
Vigorous (>6.0 MET)	−0.078	−0.001	−0.074
	0.175	0.984	0.195
Activity type			
Household/Caregiving	0.124	−0.212	0.170
	0.030	<0.001	0.003
Occupational	−0.035	0.409	−0.154
	0.542	<0.001	0.007
Sports/exercise	−0.120	0.136	−0.181
	0.036	0.017	0.002

### Planned Pregnancy

Of the women pregnant %76.6 (*n* = 233) had planned pregnancy. The mean total activity scores of the pregnant women with unplanned pregnancy were significantly higher (220.95 ± 114.92; 118.19 ± 104.74 MET-hours/week, respectively) (*p* = 0.005). There was no significant effect of planned pregnancy on sedentary and vigorous activities (*p* > 0.05). Light and moderate activity median scores of women with unplanned pregnancy were significantly higher (111.6 (6.4–268.3); 84.7(0.0–282.5) MET-hours/week) (*p* < 0.001) and (46.7(0.0–327.6); 33.9(0.0–326.2) MET-hours/week) (*p* = 0.010), respectively.

Household/caregiving activity median scores of women with unplanned pregnancy were significantly higher (131.5 (6.4–440.7); 84.7 (0.0–470.8) MET-hours/week, respectively) (*p* < 0.001). In sports/exercise activities, the median scores women with planned pregnancy were significantly higher (7.7(0.0–176.8); 2.7(0.0–55.9) MET-hours/week, respectively) (*p* = 0.029). There was no significant effect of the planned pregnancy on occupational activities (*p* > 0.05).

### High Risk Pregnancy

The mean total activity scores of women with a high risk pregnancy in previous pregnancies, were significantly higher (254.84 ± 126.73; 200.54 ± 100.85 MET-hours/week, respectively) (*p* = 0.007). The median sedentary activity scores were also higher in women with a high risk pregnancy history (33.3 (3.7–96.1); 21.5 (3.7–151.0) MET-hours/week, respectively) (*p* = 0.048).

Moderate activity median scores of women with a high risk pregnancy history were also significantly higher (60.6 (7.0–293.6); 43.8 (0.0–326.2) MET-hours/week, respectively) (*p* = 0.009). There was no significant effect of history of high risk pregnancy on activity types (*p* > 0.05).

### Trimesters

There were 56 (18.4%) women from the first trimester, 113 (37.2%) from the second trimester, and 135 (44.4%) from the third trimester. 1st, 2nd and 3rd trimester mean total activity scores of pregnant women were 202.57 ± 114.25, 205.69 ± 108.05, 184.81 ± 104.82 MET-hours/week, respectively. There was no significant difference in total activity scores between trimesters (*p* > 0.05). The median sedentary activity scores of pregnant women in the first trimester were significantly higher than those in the third trimester (37.5 (1.9–131.3); 24.9 (1.9–153.3) MET-hours/week, respectively) (*p* = 0.015). No significant relationship in sedantary activity scores was found between first and second trimesters (*p* > 0.05).

While there was no significant difference between the light, moderate and vigorous activity scores of the women in different trimesters (*p* > 0.05), the mean occupational activity scores of the women in the first trimester were significantly higher than those in the third trimester (27.77 ± 55.22; 7.98 ± 24.99, MET-hours/week respectively) (*p* = 0.011). There was no significant relationship between pregnancy trimester and other activity types (*p* > 0.05).

### Presence of Child

Means of total activity scores of pregnant women having children were significantly higher than those without children (212.82 ± 110.04, 169.45 ± 99.30 MET-hours/week, respectively) (*p* < 0.001). Median sedentary activity scores of the pregnant women who did not have children were significantly higher (40.3 (1.9–158.0), 21.0 (3.7–125.8) MET-hours/week, respectively) (*p* < 0.001).

Light and moderate activity median scores of pregnant women with children were significantly higher (111.0 (0.0–266.9), 66.7 (25.4–282.5); 48.2 (0.0–327.6), 22.1 (0.0–326.2) MET-hours/week, respectively) (*p* < 0.001 for both).

Household/caregiving activity median scores of pregnant women with children were significantly higher (136.2 (0.0–470.8), 56.9 (31.6–466.2, MET-hours/week respectively) (*p* < 0.001). Mean and median scores of occupational activity and sports/exercise activity scores of the women who did not have children were significantly higher (28.13 ± 47.97, 6.33 ± 26.43); (11.2 (0.0–176.8), 3.9 (0.0–113.1) MET-hours/week, respectively (*p* < 0.001), (*p* = 0.008).

### Environmental Characteristics

Seven questions from IPAQ-E scale were asked for the evaluation of the effect of environmental characteristics on physcial activity. The percentages of answers for each questions are shown in Table 4.

**TABLE 4:** Questions of international physical activity environment module (Istanbul, Türkiye. 2023).

	I Agree n (%)	I do not know/I am not sure n (%)	I Disagree n (%)
1. Places like shops, stores, markets, etc. are within 10–15 min walking distance from my house	281 (92.4)	0 (0.0)	23 (7.6)
2. Public transportation stops (bus, train, tram) are within 10–15 min walking distance from my house	278 (91.4)	2 (0.7)	24 (7.9)
3. There are free or low-cost hiking trails, bike paths, recreation centers, public swimming pools, etc. in my neighborhood	232 (76.3)	15 (4.9)	57 (18.8)
4. The pavements in my neighborhood are wide, well-maintained and suitable for walking	212 (69.7)	26 (8.6)	66 (21.7)
5. Due to the crime rate in my neighborhood it is not safe to walk during the day	50 (16.4)	54 (17.8)	200 (65.8)
6. In my neighborhood, there are open sports fields (such as basketball and volleyball courts) that can be used free of charge	163 (53.6)	31 (10.2)	110 (36.2)
7. In my neighborhood, there are traffic signs and pedestrian crossings on crowded streets	249 (81.9)	15 (4.9)	40 (13.2)

For each of the IPAQ-E questions, those answered as I do not know/not sure and those answered as I disagree constitute a group; those answered as I agree were evaluated as a second group. The PPAQ scores of these two groups created separately for each question were compared.

The mean sedentary activity scores of pregnant women whose home were within 10–15 min of walking distance from markets, etc. significantly higher (37.89 ± 30.95, 29.07 ± 32.19 MET-hours/week, respectively) (*p* = 0.037). No significant relationship was found with other PPAQ scores (*p* > 0.05).

The sports/exercise activity median scores of the women living in a neighborhood existing free or low-cost walking trails, bike paths, etc. were significantly lower (5.8 (0.0–113.1), 9.1 (0.0–176.8) MET-hours/week, respectively) (*p* = 0.016). No significant relationship was found with other PPAQ scores (*p* > 0.05).

Pregnant women who thought that the sidewalks in the neighborhood are wide and suitable for walking had significantly higher mean total activity scores (228.36 ± 119.58,181.74 ± 99.46 MET-hours/week, respectively) (*p* < 0.001). The median sedentary activity, light activity and moderate activity scores of them were also significantly higher (35.4 (1.9–151.0), 25.6 (1.9–158.0); 101.9 (20.7–277.2), 90.3 (0.0–282.4); 38.6 (0.0–273.7), 34.0 (0.0–327.6), MET-hours/week respectively) (*p* < 0.001, *p* = 0.048, *p* = 0.028). There was no significant relationship between the structure of pavements and other PPAQ scores (*p* > 0.05).

The median sedentary activity scores of pregnant women who thought that it is not safe to walk during the day due to the crime rate in the neighborhood were significantly lower (21.0 (1.9–158.0), 29.9 (1.9–153.3) MET-hours/week) (*p* = 0.007). There was no significant relationship between other PPAQ scores and the perception of crime rates (*p* > 0.05).

The median sedentary activity scores of pregnant women who thought that there are traffic signs and pedestrian crossings on crowded streets in the neighborhood were significantly lower (26.3 (1.9–158.0), 34.5 (3.7–151) MET-hours/week, respectively) (*p* = 0.008). No significant relationship was found with other PPAQ scores (*p* > 0.05).

There was no significant relationship between PPAQ scores and being within 10–15 min walking distance of public transportation stops and presence of free open sports complex in neighborhood (*p* > 0.05).

### Multivariate Analysis

A total activity score of median or higher was considered as high total activity level. The high total activity score was considered as the dependent variable and high total acitivity was coded as 1, low total activity was coded as 0 in the multivariate analysis. Binary logistic regression models were created with variables that had a significant relationship with total activity in univariate analyzes. The variables of working status, presence of children, previous high-risk pregnancy, planned pregnancy, suitability of pavements were included in the analysis as independent variables and three variables remained in the model in the last step. Women who have been working, had children and high risk pregnancy before had significantly higher total activity scores (Table 5).

**TABLE 5:** Multivariate analysis of participants’ sociodemographic, pregnancy-related conditions and environmental features with total activities (Istanbul, Türkiye. 2023).

N:304		*p*-value^a^	OR	95% confidence interval	
				Lower	Upper
Working status	Not working (referance)				
	On maternity leave	0.974	0.979	0.276	3.472
	Working	0.001	9.712	2.388	39.503
Presence of child	No (referance)				
	Yes	0.026	4.532	1.195	17.194
Previous high-risk pregnancy	No (referance)				
	Yes	0.003	4.941	1.705	14.314

^a^ Binary logistic regression test (backward LR method) was used.

Variables included in the model: Working status, presence of children, previous high-risk pregnancy, planned pregnancy, suitability of pavements.

### Qualitative Findings

Physical activity should be provided during pregnancy. For this reason, learning the perceived benefits and barriers of physical activity of pregnant women will ensure that interventions that will promote physical activity during pregnancy can be more focused and will increase the success rates of interventions. Thus; there is a need for an in-depth ecvaluation of the situations such as the physical complaints related to pregnancy, the benefits of physical activity, the reasons for decreased physical activity during pregnancy, and how physical activity is affected during the pandemic period. In this way, in-depth interviews were also conducted with 14 pregnant women in the study. The characteristics of these women are summarized in Supplemantary Table S1.

Three different themes were created: Pregnancy-Related Complaints, Physical Activity During Pregnancy, Concerns and Measures Regarding COVID-19. The themes, categories and the frequency of the codes that make up the categories are shown in Table 6.

**TABLE 6:** Themes, categories, and frequency of codes that make up the categories (Istanbul, Türkiye. 2023).

Themes	Categories	Frequency (code)
Pregnancy-Related Complaints	Physical Findings in Pregnancy	56
	Benefits of Physical Activities During Pregnancy on Physical Findings	16
	Total	72
Physical Activity During Pregnancy	Physical Activity Before Pregnancy	30
	Reasons to Be Less Active During Pregnancy	37
	Persons Accompanying Physical Activity	28
	Purpose of Physical Activity	37
	Total	132
Concerns and Measures Regarding COVID-19	Concerns About COVID-19	21
	Leaving Home After COVID-19	26
	Participation in Social Activities	27
	Total	74

#### Theme 1: Pregnancy-Related Complaints

The categories mentioned about pregnancy-related complaints were “Physical Findings in Pregnancy” and “Benefits of Physical Activities During Pregnancy on Physical Findings”.

##### Category 1: Physical Findings in Pregnancy

Pregnant women mostly mentioned that they experienced nausea and vomiting and low back pain during pregnancy, respectively. Constipation and sleepiness were other complaints mentioned. The examples of the statements of pregnant women are below:

*“I have nausea and vomiting and it still continues. There is constant nausea during the day due to pregnancy-related hormones. In the first weeks, it occured every day, now it has decreased, but it is still exists.”* (P1).

*“I had low back pain, after the fifth month, the baby became heavier, and I am thin, and I did not gain weight in the first months. I have had a lot of back pain and I still have extreme back pain. I can't walk straight because of back pain."* (P12).

##### Category 2: Benefits of Physical Activities During Pregnancy on Physical Findings

Pregnant women mentioned that physical activity is mostly good for back pain. Improving nausea, constipation and sleep were the other benefits of women experienced. Some statements regarding the change of physical findings with physical activity are given below:

*“I can say that when I sit too much, my back hurts more. It is more difficult for me to stand up when I sit too much. When you move, you feel more comfortable.”* (P3).

*“Getting fresh air can be good for my nausea. It is decreasing, it is getting lighter.”* (P1).

*“I feel that it is more comfortable as I move, as I walk. It is even that I go to the bathroom after the walks.”* (P4).

“Walking helps me to sleep better at night. It allows me to sleep uninterrupted.” (P3).

#### Theme 2: Physical Activity During Pregnancy

The categories mentioned about Physical Activity in Pregnancy were “Physical Activity Before Pregnancy,” “Reasons for Being Less Active During Pregnancy,” “Persons Accompanying Physical Activity” and “Purpose of Physical Activity.”

##### Category 1: Physical Activity Before Pregnancy

Participants mentioned that they were less active more frequently during pregnancy. Eight women stated that they were less active during pregnancy. Some pregnant women mentioned that their movements slowed, and they could not achieve the old respiratory condition. Some of the statements are below:

*“I am less active and more tired during pregnancy. I move slower. I have difficulty in breathing…..I can't be very active unfortunately..”* (P3).

*“I can't climb stairs, I breath fast. ….And when I do housework, I have diffuculty in breathing as if I have done a huge work..….When I have diffuclty in breathing, I stop immediately. I'm slowing down."* (P11).

Activity left during pregnancy was another topic mentioned. Two women gave up cycling and dancing activities, respectively, when they were aware of their pregnancies.

##### Category 2: Reasons for Being Less Active During Pregnancy

As more than half of the women mentioned they were less active during pregnancy, we asked also the reasons for this situation. The most common reason was fatigue. Lack of time, low back pain, nausea were other reasons for decreased physical activity that women said.

*“….I get tired quickly. I don't go out for walks like I used to. I need to sit down immediately. That's why there's fatigue."* (P6).

*“I can't spare extra time for sports. My daughter is very dependent on me….I spend time with my daily work….I just can't go out for a walk…..Actually I will go out if I have time”* (P6).

##### Category 3: Persons Accompanying Physical Activity

The pregnant women mentioned their spouses, neighbors-friends, family-relatives as the accompanying person to physical activity, in order of frequency.

##### Category 4: Purpose of Physical Activity

The participants mostly mentioned that the purpose of physical activity is to improve their mental health. The improvement of physical health and pregnancy complaints, weight control, a healthier pregnancy process and delivery, improved health of the baby were other purposes.

*“Exercise relaxes me physically….Walking is also good, getting air is good, I feel better when I walk. It is also good mentally, so walking is good for that reason.”* (P2).

#### Theme 3: Concern and Measures Regarding COVID-19

The concepts mentioned under the theme of Concern and Measures about COVID-19 are “Concerns About COVID-19,” “Leaving Home After COVID-19” and “Participation in Social Activities.”

##### Category 1: Concerns About COVID-19

Participants often mentioned that their concerns about COVID-19 increase during pregnancy. In addition, the participants mentioned that the news about COVID-19 worried them.

*“Well, if I wasn't pregnant, I guess it wouldn't be a concern…….My anxiety increased when I was pregnant.”* (P4).

*“I don't want to hear it, I get depressed. My daughter is studying at an university outside the province, I am worried about her. I don't want to follow the news....”* (P7).

##### Category 2: Leaving Home After COVID-19

Participants often mentioned that going out to the necessary areas such as the market and hospital did not change compared to before the pandemic. Less women mentioned that going out for a necessary condition has decreased due to the pandemic.

##### Category 3: Participation in Social Activities

Unlike the going out to the necessary areas, participants frequently mentioned that they decreased their social events during the pandemic period. Less women said there was no change about the participation in social environments.

*“Unless there is a special situation, we have reduced it....if I'm bored, I meet always outside the home. I have also reduced the frequency of indoo meetings.”* (P2).

## Discussion

In this study, physical activity levels and related factors of pregnant women were evaluated. Pregnant women mostly engaged in housework/caregiving and light-intensity activities. In studies conducted in Turkey and other countries, participants engaged mostly in light-intensity activities and housework/caregiving activities, similarly [25–28].

In our study, as the age of pregnant women increases, sedentary activities and sports/exercise activities decreases significantly. Household/care activities were higher in women in higher ages. There are many studies examining the effect of maternal age on physical activity during pregnancy. According to some studies; as maternal age increases, higher exercise levels are observed [29, 30]. In different studies, younger maternal age is found to be associated with higher physical activity levels [9, 31]. Differences between study results may be due to cultural and social differences in societies that change with age.

The total activity score of women was highest in the 2nd trimester and lowest in the 3rd trimester in our study. This may be due to the increase in self-confidence and compliance with pregnancy in the second trimester compared to the first periods of pregnancy, and the decrease in pregnancy-related symptoms such as fatigue and nausea. The decrease in physical activity in the last trimester may also be due to the weight gain and the associated decrease in respiratory capacity, as stated in the qualitative part of our study. Similar to our results; Tosun et al. reported that the physical activity levels of the pregnant women in the first and third trimesters were similar and women increased the level of all physical activity types in the second trimester [32].

The physical environmental conditions of the neighbourhood can affect the level of physical activity [33]. According to our study, total activity scores and activity intensity scores excluding vigorous activity, and occupational activity scores of pregnant women who reported that the pavements in their neighborhood are wide, well-maintained and suitable for walking were significantly higher. Similar to our study, being close to environments suitable for physical activity such as suitable pavements, parks and playgrounds, indoor or outdoor sports halls can make it easier to exercise [34, 35].

Physical activity is recommended in pregnant women and has significant benefits for pregnant women [2]. Despite all the known benefits, women reduce or stop their physical activities during pregnancy [36]. According to the study of Ko et al., pregnant women decreased their physical activity by 31% compared to the pre-pregnancy period [37]. Participants in our study also mentioned that they were often less active and the reasons for being less active, were weakness and fatigue, lack of time, low back pain and nausea. Similarly a study reported, pregnant women tend to engage in physical activity less during pregnancy due to reasons such as fear of harm to their babies, pregnancy-related complaints, lack of motivation and time [36]. Being able to identify the factors that prevent physical activity during pregnancy will also guide the focus of interventions to be planned in this regard.

Since our study was carried out during the pandemic period, the measures (quarantine, social restriction, etc.) taken may also have contributed to reducing pregnant women’s physical activities. In a study conducted in our country; the physical activities of adults before and during the pandemic in different cities, including Istanbul, were compared. While the total activities of non-pregnant women decreased during the pandemic period compared to the pre-pandemic period; there was also an increase in sedantary times [38]. Another recent study indicates that more than half of the non-pregnant women in our country were inactive [39]. While it is expected that the physical activity levels of all individuals will be adversely affected by the pandemic measures; it is also expected that pregnant women are also affected as a susceptible group. It is important to identify that women are less active during pregnancy in our country, in order to develop more planned interventions. In this way, we have conducted our study. Inadequate physical activity during pregnancy is a wide-ranging problem that concerns many individuals and institutions; especially the health system, healthcare professionals and institutions providing pregnancy care, policymakers, and institutions about environmental regulations promoting physical activity. All these people and institutions should be alert about the low level of physical activity during pregnancy and work to make arrangements in this regard.

In our qualitative part of the study, the pregnant women mostly mentioned that the aim of physical activity is to improve their mental health during pregnancy. The other factors which guide women to physical activity are to improve the physical health and pregnancy complaints, to supply weight control and a healthier pregnancy process including delivery and to improve baby’s health. In interventions targeting to increase physical activity, pregnant women should be encouraged to engage in physical activity based on these factors. Pregnant women should also be informed about other lesser-known benefits of physical activity.

In a qualitative study, pregnant women mentioned that they experienced fear and anxiety during the COVID-19 period. They stated that being pregnant was one of the most important factors on this fear and anxiety they experienced [15]. In the same study, pregnant women mentioned that they reduced their routine daily activities such as walking and meeting with their families [15]. In our study, the majority of pregnant women mentioned that their concerns about COVID-19 increased during pregnancy. Moreover, the majority of pregnant women reduced their participation in social activities in our study during pandemic period. Since this situation can affect the mental wellbeing of pregnant women, it may cause low motivation, which is one of the important obstacles for physical activity in pregnant women [40].

### Limitations and Strengths

Evaluation of physical activity with a questionnaire (subjective method) is one of the limitations of our study. In addition, the percentage of pregnant women in the first trimester is low. This may cause a limitation for the interpretation of the results related to the pregnancy trimesters. Since our study was conducted in a hospital in a single district; the majority of the participants may be residing in the same district or a district with similar environmental characteristics. It may cause a limitation in evaluation the relationship between the environmental characteristics and physical activity. Since perceived environmental characteristics by participants were evaluated instead of an objective measure; another limitation in our study may be exist. One of the strengths of our study is that there are few studies in the literature on physical activity behaviors of pregnant women. In addition; sociodemographic variables, obstetric characteristics, environmental conditions were evaluated in our study with qualitative and quantitative methods. A broad perspective was formed with help of our study results.

### Conclusion

Pregnant women who have been working, had children and high risk pregnancy before had significantly higher total activity scores. More than half of the pregnant women mentioned that they were less active during pregnancy. Physical complaints such as weakness, fatigue, low back pain, nausea and lack of time were the reasons for being less active. To increase the physical activity levels, there is a need for interventions target especially pregnant women who are working, have children and high risk pregnancy before. Sports activities may be more encoureged as we found pregnant women mostly engaged in household activities.
