# Supplementary material for: Evaluation of Physical Activity Level and Related Factors in Pregnancy During the COVID-19 Period
Source: Int J Public Health. 2023 May 5;68:1605800. doi: 10.3389/ijph.2023.1605800 (PMC10196054; doi:10.3389/ijph.2023.1605800)
Supplement: Supplementary file 1 [file Table1.DOCX]

**Supplementary Table 1:** Sociodemographic characteristics of the participants (Istanbul, Turkey. 2023).

|  | Age | Gestational week | Pregnancy Trimester | Number of Children |
| --- | --- | --- | --- | --- |
| **P1** | 26 | 12 | 1. | - |
| **P2** | 28 | 14 | 2. | - |
| **P3** | 29 | 30 | 3. | - |
| **P4** | 31 | 34 | 3. | 1 |
| **P5** | 30 | 34 | 3. | - |
| **P6** | 29 | 23 | 2. | 1 |
| **P7** | 32 | 30 | 3. | 2 |
| **P8** | 29 | 10 | 1. | 1 |
| **P9** | 29 | 31 | 3. | - |
| **P10** | 37 | 34 | 3. | 2 |
| **P11** | 26 | 26 | 2. | 1 |
| **P12** | 28 | 34 | 3. | 1 |
| **P13** | 35 | 25 | 2. | 2 |
| **P14** | 37 | 23 | 2. | 1 |
